# Trapped sweat in basketball uniforms and the effect on sweat loss estimates

**DOI:** 10.14814/phy2.13463

**Published:** 2017-09-28

**Authors:** Lindsay B. Baker, Adam J. Reimel, Bridget C. Sopeña, Kelly A. Barnes, Ryan P. Nuccio, Peter John D. De Chavez, John R. Stofan, James M. Carter

**Affiliations:** ^1^ Gatorade Sports Science Institute Barrington Illinois; ^2^ PepsiCo, Inc. Barrington Illinois

**Keywords:** Basketball protocol, intermittent exercise, team sports

## Abstract

The aims of this study were to determine: (1) trapped sweat (TS) in basketball uniforms and the effect on sweat loss (SL) estimates during a laboratory‐based basketball simulation protocol; (2) the impact of exercise intensity, body mass, age, and SL on TS; and (3) TS during on‐court training to assess the ecological validity of the laboratory‐based results. Twenty‐four recreational/competitive male basketball players (23 ± 10 years, 77.0 ± 16.7 kg) completed three randomized laboratory‐based trials (Low, Moderate, and High intensity) consisting of 150‐min intermittent exercise. Eighteen elite male players (23 ± 4 years, 92.0 ± 20.6 kg) were observed during coach‐led, on‐court training. Nude and clothed body mass were measured pre and postexercise to determine TS. Data are mean ± SD. There was a significant effect of intensity on SL and TS (*P* < 0.001, Low<Moderate<High, ANOVA). During Low, subjects lost 1.10 ± 0.59 kg sweat and TS was 0.11 ± 0.15 kg (8.0 ± 5.1% SL). During Moderate, subjects lost 1.60 ± 0.56 kg sweat and TS was 0.21 ± 0.21 kg (11.6 ± 6.3% SL). During High, subjects lost 2.12 ± 0.66 kg sweat and TS was 0.38 ± 0.28 kg (16.0 ± 7.4% SL). Multiple regression and partial correlation analysis suggested TS was significantly related to SL (*P* < 0.0001; partial *r* = 0.81–0.89), whereas the contributions of body mass (*P* = 0.22–0.92) and age (*P* = 0.29–0.44) were not significant. TS during on‐court training was 0.35 ± 0.36 kg, which was associated with a 14.1 ± 11.5% underestimation in SL, and was not statistically different than laboratory‐based results (*P* = 0.59). Clothed body mass measurements should be used with caution, as TS is highly variable and can cause a significant underestimation in SL in athletes with high sweating rates.

## Introduction

Measuring athletes' sweat loss is an important component of determining personalized fluid replacement strategies to prevent the potential negative effects of significant fluid imbalances on health and performance (Sawka et al. 2007; Hew‐Butler et al. 2015; Thomas et al. 2016). Sweat loss and fluid balance are most commonly estimated by measuring changes in body mass during exercise (Armstrong 2007). While nude body mass is the most accurate method, it is primarily limited to laboratory‐based studies. When testing is conducted in the field, athletes' body mass is often measured while they are wearing some clothing or sports uniform (Cheuvront and Haymes 2001). While this is a more practical method, any sweat that is absorbed or trapped in the clothing can confound the sweat loss calculations. However, little research has been conducted to determine how much sweat is trapped in sports uniforms and how it impacts calculated sweat loss at varying exercise intensities (Cheuvront et al. 2002; Noonan and Stachenfeld 2012; O'Neal et al. 2012). Such data could be instrumental in the development of best practice recommendations for measuring sweat losses in the field (Cheuvront and Kenefick 2017).

No studies, to our knowledge, have measured trapped sweat in basketball; where high‐intensity bouts of exercise can lead to high sweat loss and therefore the potential for trapped sweat may also be substantial. For example, in elite male youth players, mean sweating rates were reported to be ~1.0–1.4 L/h during training and ~1.6 L/h during competition (Broad et al. 1996). Furthermore, mean sweating rates of ~1.4‐1.6 L/h were observed in male college players during game play (Afman et al. 2014). Similarly, in professional basketball players, mean sweat losses were as high as 2.4 L during 40‐min exhibition games (Osterberg et al. 2009). These high sweating rates may be due, in part, to the large body size of basketball players as well as the intensity of training and competition. In addition, since basketball is played indoors in temperate conditions, the relatively low air flow may lead to less sweat evaporation and therefore more trapped sweat compared with outdoor sports.

The primary aim of this study was to measure the amount of sweat that becomes trapped in basketball clothing during training and to determine the error in sweat loss calculations associated with clothed body mass measurements. Secondarily, we aimed to determine how exercise intensity, exercise duration, body mass, age, and sweat loss impact the amount of trapped sweat in basketball uniforms. To maintain systematic control of exercise intensity and standardize other factors (e.g., air temperature, relative humidity, clothing ensemble), a laboratory‐based basketball‐simulation protocol was used. Therefore, another aim of the study was to measure trapped sweat in a sample of basketball players during actual on‐court training to assess the ecological validity of the laboratory‐based results. It was hypothesized that trapped sweat would be similar between laboratory and field‐based observations and that trapped sweat would be associated with significant underestimations in sweat loss.

## Methods

### Subjects

A total of 42 male basketball players volunteered to participate in this study. The laboratory‐based testing was completed by 24 players, whom were currently participating in their junior or senior high school programs or local recreational leagues (23 ± 10 years, 77.0 ± 16.7 kg). Eighteen participants, whom were high school, college, or professional National Basketball Association players completed the on‐court testing (23 ± 4 years, 92.0 ± 20.6 kg). This study was approved by the Sterling Institutional Review Board (Atlanta, GA) for the protection of human study participants. Participants and their parent/guardian were informed of the experimental procedures and associated risks before providing written informed consent.

### Experimental procedures during laboratory‐based testing

The 24 subjects participating in the laboratory‐based study completed one preliminary visit to determine body mass and height (180.2 ± 9.8 cm) as well as maximal rate of oxygen consumption (49.7 ± 5.8 mL/kg/min), and maximal heart rate (186 ± 10 bpm) during a graded exercise test to exhaustion on a treadmill. After the preliminary visit, each subject completed three experimental trials in random order with at least 3 days, but not more than 14 days between trials. The trials differed only by the intensity of the intermittent exercise protocol; that is Low, Moderate, and High intensity (see below for more details). Subjects reported to the laboratory after having fasted for 2 h and refrained from strenuous exercise, caffeine, and alcohol for 24 h. Subjects were asked to drink 500 mL of water 2 h before the scheduled start of their trial. At baseline the 24‐h diet and 1‐week activity logs were reviewed to confirm consistency between trials. After voiding their bladders, subjects had baseline body mass measured, first while nude and then while wearing the basketball uniform provided to them by the investigators.

During each trial, subjects wore the same standard game uniform, consisting of jersey top and shorts (555 g; 92% nylon, 8% spandex; Russell Athletic), compression tank top (127 g; 90% polyester, 10% spandex; Under Armour), compression shorts (67 g; 90% polyester, 10% spandex; Nike), compression sleeve on one arm (31 g; 80% nylon, 20% spandex; McDavid) and both knees (47 g; 80% nylon, 20% spandex; McDavid), head sweat band (20 g; 100% polyester; Under Armour), and crew socks (86 g; 62% polyester, 21% nylon, 15% cotton, 2% spandex; Nike). The subjects wore the same pair of their own basketball shoes for each trial.

The exercise protocols were designed to simulate the physical demands of basketball training or play based on previous literature (McInnes et al. 1995; Ben Abdelkrim et al. 2007, 2010; Narazaki et al. 2009; Scribbans et al. 2015; Puente et al. 2016). The face validity of the basketball‐simulation protocols were confirmed via feedback from basketball players and coaches. Each trial consisted of 150 min (5 × 30‐min bouts) of intermittent exercise in a temperate room (22.5 ± 0.8°C, 62 ± 7% relative humidity, ambient air flow of ~0.5 m/sec). This protocol duration was chosen since the total “clock time” of practices and games can be up to ~150 min. The Low‐, Moderate‐, and High‐intensity trials differed in the percentage of time spent performing strenuous activities such as running (4.0 m/sec), sprinting (5.3 m/sec), and drills (lateral slides, jumping, footwork drills, pushups, basketball passes) versus time spent doing light activities such as standing, walking (1.5 m/sec), and jogging (3.1 m/sec). The ratio of time spent performing strenuous versus light activity was 11%:89% for Low, 26%:74% for Moderate, and 45%:55% for High. In general, the three levels of exercise intensity were designed to simulate the demands of a walkthrough/shoot‐around (Low), a moderate‐intensity practice (Moderate), and a high‐intensity practice or game (High).

Twenty‐five minutes into each bout of exercise, there was a ~5–10‐min break for subjects to void their bladder (urine sample was collected and subsequently weighed) followed by body mass measurements with clothing and then nude. Subjects were instructed to towel‐dry before each body mass measurement. During each trial, subjects were provided with fluid at 15‐min intervals to replace ~80% of fluid losses (based on body mass losses). The amount of fluid intake was determined by weighing the drink bottles before and after consumption using digital scales (to the nearest 0.01 g; Mettler Toledo, PG6002‐S). Heart rate (HR; Polar) and ratings of perceived exertion (RPE; Borg 6‐20 scale) were recorded every five min throughout the 150‐min protocol. Before and after exercise, subjects rated their muscle fatigue, tiredness, effort, and physical demand on a 100‐point visual analog scale (VAS). For each question on the VAS, the responses ranged from “none” (0) to “very/severe” (100).

### Experimental procedures during on‐court testing

To determine trapped sweat in basketball clothing during actual on‐court training, observational data collection was conducted during indoor (21.9 ± 0.2°C, 51 ± 12% relative humidity) basketball practices (1.74 ± 0.71 h). For this part of the study, players were tested each during one practice. The practice uniform was issued by the team and generally consisted of synthetic practice shorts, jersey top, compression shorts, socks, and shoes. Five of the 18 players also wore compression knee and/or arm sleeves. The practice intensity was rated as low by six players, moderate by seven players, and high by five players.

Before exercise, athletes voided their bladder and then had body mass measured on a digital platform scale (to the nearest 0.01 kg; Rice Lake IQ+355, Sartorius Combics 2 CISL2‐U, and Detecto DR400‐750), first while wearing their full practice uniform and then while nude or wearing only compression shorts. The body mass scale varied by testing site (i.e., field vs. laboratory) but was consistent within each player. Ad libitum fluid intake during exercise was determined by weighing the drink bottles before and after consumption using compact digital scales (to the nearest 1 g; Ohaus CS2000). Water and sports drink of the athlete's preference, in bottles labeled specifically for each athlete, were available throughout the duration of the practice. Athletes were instructed to avoid spitting, spilling, or pouring fluid from the bottles. If athletes needed to urinate during practice they were given a preweighed container and asked to collect all urine for subsequent weighing. After completion of practice, the athletes towel‐dried themselves and then had their body mass measured with the same scale and while wearing the same clothes as the preexercise clothed body mass assessment. Finally, the players removed all clothing (except their compression shorts in some cases), towel‐dried, and then had their body mass measured.

### Calculations

Trapped sweat was calculated as the difference between pre and postexercise uniform mass, where:

$$\text{Uniform}\;{\text{Mass}}_{\mathrm{preexercise}}=\text{Clothed Body}\;{\text{Mass}}_{\mathrm{preexercise}}-\text{Nude Body}\;{\text{Mass}}_{\mathrm{preexercise}}$$

$$\text{Uniform}\;{\text{Mass}}_{\mathrm{postexercise}}=\text{Clothed Body}\;{\text{Mass}}_{\mathrm{postexercise}}-\text{Nude Body}\;{\text{Mass}}_{\mathrm{postexercise}}$$

Sweat loss was calculated both with and without corrections for trapped sweat, metabolic mass loss, and respiratory water loss as follows:

Uncorrected sweat loss=Δclothed BM+fluid intake−urine output

where ∆ clothed BM was the change in clothed body mass from pre to postexercise (kg), fluid intake was the mass of fluid consumed during exercise (kg), and urine output was the mass of urine collected between the pre and postexercise body mass measurements (kg).

Partially corrected sweat loss=Δnude BM+fluid intake−urine output

where ∆ nude BM was the change in nude body mass from pre to postexercise (kg).

Corrected sweat loss=Δnude BM+fluid intake−urine output−MML−RWL

where MML was metabolic mass loss and RWL was respiratory water loss. MML and RWL were calculated using published equations (Mitchell et al. 1972), for which oxygen consumption and respiratory quotient were estimated based on corresponding heart rate measurements between the trial and the graded exercise test (preliminary visit). All calculations were performed for the laboratory‐based testing. For the on‐court testing, only trapped sweat, uncorrected sweat loss, and partially corrected sweat loss were calculated. There was not enough information regarding oxygen consumption to estimate metabolic mass loss and respiratory water loss to calculate corrected sweat loss.

### Statistical analyses

Analyses were carried out using Statistical Analysis Software version 9.4 (SAS Institute; Cary, NC), Minitab 17 Statistical Software (Minitab Inc.; State College, PA), and XLSTAT 2016 (Addinsoft; New York, NY). Based on previous pilot work it was determined that to detect a mean ± SD difference of 0.10 ± 0.16 kg of trapped sweat between trials a sample size of 23 subjects would be needed.

A repeated measures (RM) one‐way analysis of variance (ANOVA) was performed to determine the effect of exercise intensity on HR, RPE, and VAS ratings as well as uncorrected sweat loss, partially corrected sweat loss, corrected sweat loss, metabolic mass loss, and respiratory water loss. To determine the effect of exercise intensity and duration on trapped sweat a RM two‐way ANOVA was conducted. A RM one‐way ANOVA was used to determine differences between exercise intensities in the percent contribution of metabolic mass loss, respiratory water loss, and trapped sweat to the sweat loss error. For all ANOVAs, a Tukey's HSD post hoc analysis was conducted where main effects were found. Multiple regression and partial correlation analyses were conducted to determine the relation between age, body mass, and corrected sweat loss versus trapped sweat. To compare mean trapped sweat results in the field versus laboratory a two independent samples t‐test was conducted.

Shapiro–Wilk tests were conducted to assess normality of the distribution of the data. In instances of deviation from normality, data were natural‐log transformed prior to analyses; this included: percent contribution of metabolic mass loss and respiratory water loss to the sweat loss error. For variables in which log transformation did not normalize the distribution of the data, nonparametric as well as parametric analyses were conducted; these included: trapped sweat and sweat loss (corrected, partially corrected, and uncorrected). The conclusions were not different between parametric and nonparametric analysis, thus the data are presented in its original form and the parametric results are shown in the results section for uniformity and clarity. Descriptive statistics are presented as mean ± standard deviation. The significance level for all statistical tests was set at *α *= 0.05.

## Results

### Laboratory‐based testing

The coefficient of variation for subjects' baseline body mass among the three trials was 0.7 ± 0.3% (0.2–1.2%). As expected, %HR_max_ (63 ± 6%, 75 ± 6%, and 82 ± 6%_,_ respectively) and RPE (9 ± 1, 12 ± 1, and 14 ± 1, respectively) increased progressively from Low to Moderate to High trials (*P* < 0.0001). VAS ratings of muscle fatigue (18 ± 3, 41 ± 5, and 62 ± 4, respectively), tiredness (10 ± 4, 34 ± 6, and 67 ± 4), effort (21 ± 3, 46 ± 3, and 72 ± 4), and physical demand (21 ± 4, 44 ± 4, 67 ± 5) also increased progressively from Low to Moderate to High trials (*P* < 0.0001).

Fluid balance and trapped sweat estimations during Low, Moderate, and High basketball‐simulation trials are shown in Table 1. There was a significant effect of exercise intensity on total uncorrected sweat loss, partially corrected sweat loss, metabolic mass loss, respiratory water loss, corrected sweat loss, and trapped sweat (Low<Moderate<High; *P* < 0.0001). Trapped sweat expressed as a percentage of baseline body mass was 0.12 ± 0.12% for Low, 0.25 ± 0.18% for Moderate, and 0.45 ± 0.25% for High trials.

**Table 1:** Fluid balance estimations during Low, Moderate, and High intensity basketball‐simulation trials

	Uncorrected sweat loss (kg)*	Trapped sweat (kg)*	Partially corrected sweat loss (kg)*	Metabolic mass loss (kg)*	Respiratory water loss (kg)*	Corrected sweat loss (kg)*
Low intensity	1.24 ± 0.50	0.11 ± 0.15	1.33 ± 0.57	0.11 ± 0.04	0.14 ± 0.04	1.10 ± 0.59
Moderate intensity	1.72 ± 0.41	0.21 ± 0.21	1.93 ± 0.63	0.15 ± 0.04	0.18 ± 0.05	1.60 ± 0.56
High intensity	2.13 ± 0.48	0.38 ± 0.28	2.51 ± 0.74	0.19 ± 0.05	0.21 ± 0.05	2.12 ± 0.66

Values are means ± SD. “Uncorrected sweat loss” is the change in clothed body mass adjusted for fluid intake and urine output only. “Partially corrected sweat loss” is the change in nude body mass corrected for fluid intake and urine output. “Corrected sweat loss” is the change in nude body mass corrected for fluid intake, urine output, respiratory water loss, and metabolic mass loss. *P < 0.0001, Low<Moderate<High

### Effect of trapped sweat on sweat loss calculation error

**Figure 1:**
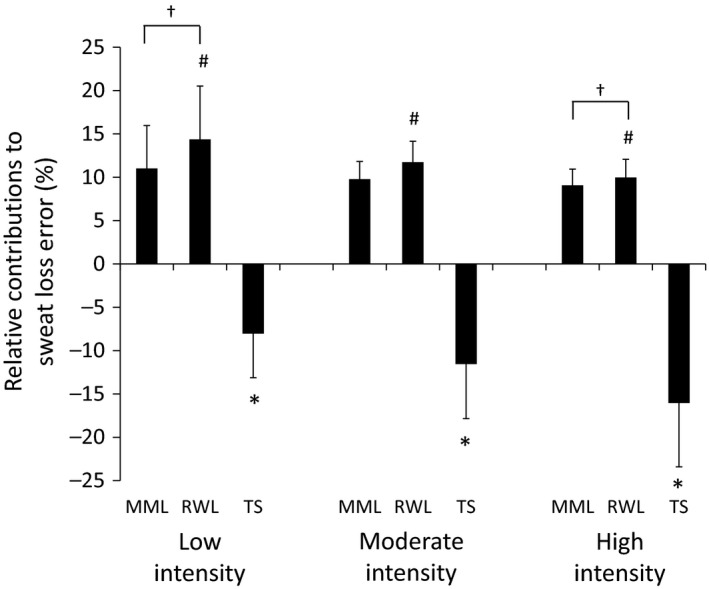
Relative contributions to sweat loss error from metabolic mass loss, respiratory water loss, and trapped sweat during the Low, Moderate, and High intensity 150‐min basketball‐simulation trials. Values are mean ± SD. MML, metabolic mass loss; RWL, respiratory water loss; TS, trapped sweat. * *P* < 0.05, Low<Moderate<High for TS; # *P* < 0.05, Low>Moderate>High for RWL; † *P* < 0.05, Low>High for sum of MML and RWL.

Figure 1 shows the relative contributions to sweat loss error from metabolic mass loss, respiratory water loss, and trapped sweat in basketball uniforms during Low, Moderate, and High basketball‐simulation trials. The underestimation in sweat loss associated with clothed body mass measurements was 8.0 ± 5.1%, 11.6 ± 6.3%, and 16.0 ± 7.4%, in Low, Moderate, and High trials, respectively. With an increase in exercise intensity, the percent contribution to the sweat loss error decreased for respiratory water loss (Low>Moderate>High; *P* < 0.05) and the sum of metabolic mass loss and respiratory water loss (Low>High; *P* < 0.05), but increased for trapped sweat (Low<Moderate<High; *P* < 0.05).

### Effect of exercise intensity and time on trapped sweat

**Figure 2:**
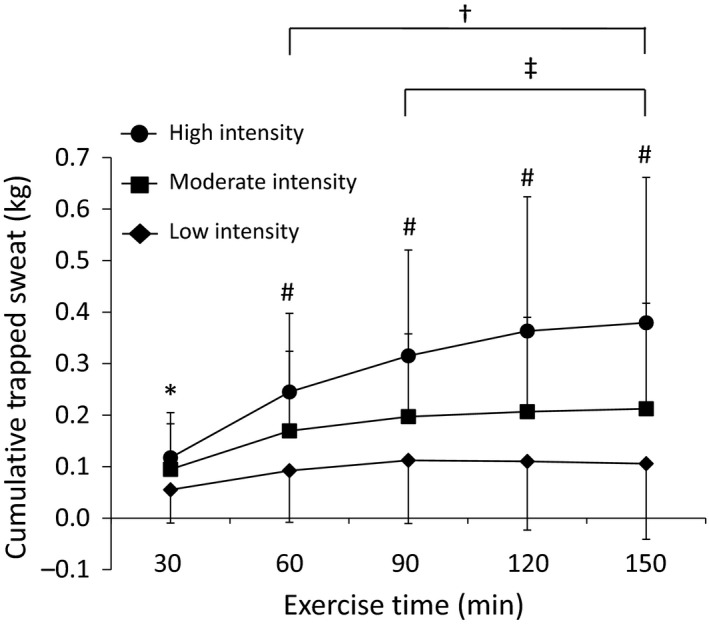
Cumulative trapped sweat at 30‐min intervals during the Low, Moderate, and High intensity 150‐min basketball‐simulation trials. Values are mean ± SD. Significant main effect of intensity (*P* < 0.0001), time (*P* < 0.0001), and significant intensity × time interaction (*P* < 0.0001). *P < 0.05, Low<Moderate and High; ^#^ *P* < 0.05, Low<Moderate<High; ^†^ *P* < 0.05, 60–150 min versus 30 min during Low, Moderate, and High trials; ^‡^ *P* < 0.05, 90–150 min versus 30–60 min during High trials.

Cumulative trapped sweat at 30‐min intervals during 150 min of Low, Moderate, and High trials is shown in Figure 2. There was a significant main effect of intensity (*P* < 0.0001), time (*P* < 0.0001), and significant intensity x time interaction (*P* < 0.0001) on trapped sweat. During the Low and Moderate trials, trapped sweat increased significantly from 30 min to 60 min (*P* < 0.05) and did not change thereafter. During the High trial, trapped sweat continued to increase from 30 min to 90 min (*P* < 0.05) before leveling off for the remainder of the 150‐min protocol. Mean ± SD cumulative trapped sweat at the end of the 150‐min protocol, expressed as a percentage of total corrected sweat loss, during the Low, Moderate, and High trials was 8.0 ± 5.1%, 11.6 ± 6.3%, and 16.0 ± 7.4%, respectively (Low<Moderate<High; *P* < 0.0001).

### Multiple regression and partial correlation analysis

Table 2 shows the bivariate correlation matrix for body mass, age, corrected sweat loss, and trapped sweat for Low, Moderate, and High trials. The matrix reveals a significant degree of collinearity among body mass, sweat loss, and trapped sweat. However, multiple regression analysis showed that at each exercise intensity, total sweat loss was the only factor that contributed significantly to the variation in trapped sweat (*P* < 0.0001), whereas the contributions of body mass (*P* = 0.92, 0.22, and 0.32 for Low, Moderate, and High trials, respectively) and age (*P* = 0.44, 0.29, and 0.32 for Low, Moderate, and High trials, respectively) were not significant. Based on the partial correlation analysis, after adjusting for body mass and age, sweat loss accounted for a significant amount of the variability in trapped sweat (partial *r* = 0.82, 0.81, and 0.89 for Low, Moderate, and High trials, respectively; *P* < 0.0001).

**Table 2:** Correlation matrix among trapped sweat, body mass, age, and corrected sweat loss for Low, Moderate, and High intensity basketball‐simulation trials

	Low intensity				Moderate intensity				High intensity			
	Trapped Sweat (kg)	Body Mass (kg)	Age (years)	Sweat Loss (kg)	Trapped Sweat (kg)	Body Mass (kg)	Age (years)	Sweat Loss (kg)	Trapped Sweat (kg)	Body Mass (kg)	Age (years)	Sweat Loss (kg)
Trapped Sweat (kg)	1				1				1			
Body Mass (kg)	0.66 *<0.001*	1			0.76 *<0.0001*	1			0.79 *<0.0001*	1		
Age (years)	−0.03 *0.90*	0.39 *0.06*	1		0.13 *0.56*	0.39 *0.06*	1		0.14 *0.54*	0.39 *0.06*	1	
Sweat Loss (kg)	0.92 *<0.0001*	0.76 *<0.0001*	0.06 *0.77*	1	0.93 *<0.0001*	0.86 *<0.0001*	0.10 *0.65*	1	0.96 *<0.0001*	0.84 *<0.0001*	0.10 *0.64*	1

Values are bivariate correlations with *P* values shown in *italics*.

**Figure 3:**
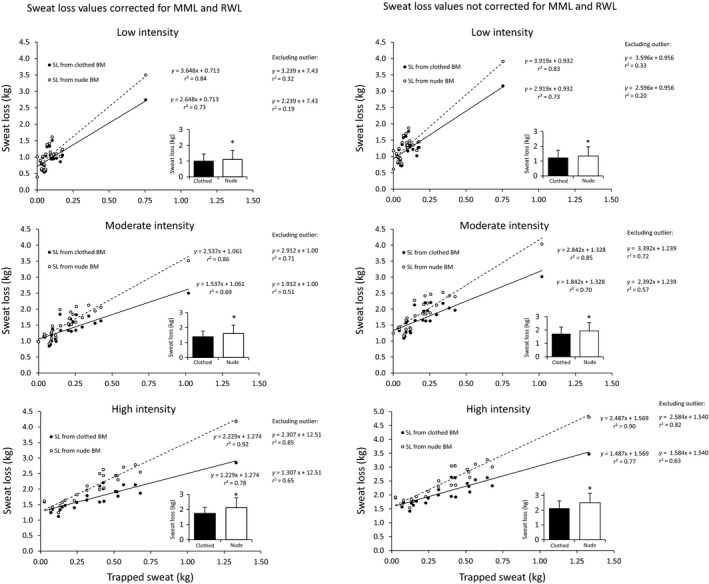
Relation between sweat loss (*y*‐axis) and the amount of trapped sweat in clothing (*x*‐axis) during the Low, Moderate, and High trials. Sweat loss data are shown both with (left column) and without (right column) corrections for metabolic mass loss (MML) and respiratory water loss (RWL). Simple linear regression equations and *r* ^2^ values are shown for each intensity level for all subjects (*n* = 24). For comparison, regression equations and *r* ^2^ values are also shown for the dataset which excludes the one outlier with high sweat losses (*n* = 23). Figure insets are the mean ± SD nude and clothed sweat losses. *P < 0.001, Clothed sweat loss < Nude sweat loss.

Figure 3 illustrates the relation between sweat loss and the amount of trapped sweat in clothing in response to Low, Moderate, and High trials. The strong linear function between nude whole body sweat loss and trapped sweat is clear across all three exercise intensities. Figure 3 builds upon the multiple regression analysis in that the observed common y‐intercept between clothed and nude body mass, in conjunction with different slopes, shows that trapped sweat produces bias that is proportional and predictable as a function of sweat loss. Histogram insets show the mean differences in sweat loss between nude and clothed body mass.

### Trapped sweat during on‐court testing

During the on‐court testing uncorrected sweat loss was 1.61 ± 0.87 kg and partially corrected sweat loss was 1.97 ± 1.13 kg. Trapped sweat during the on‐court testing was 0.35 ± 0.36 kg. Trapped sweat was not significantly different between the on‐court testing and the laboratory‐based testing for the mean of all trials (0.23 ± 0.21 kg, *P* = 0.59), the High trials (0.38 ± 0.28 kg, *P* = 0.46), and the Moderate trials (0.21 ± 0.21 kg, *P* = 0.43), but was different than the Low trials (0.11 ± 0.15 kg, *P* = 0.03). Trapped sweat expressed as a percentage of partially corrected sweat loss during the on‐court testing was 14.1 ± 11.5%. Trapped sweat expressed as a percentage of baseline body mass was 0.36 ± 0.40%.

## Discussion

The aims of this study were to determine (1) the amount of trapped sweat in basketball uniforms and the associated impact on sweat loss estimates during a laboratory‐based basketball simulation protocol; (2) the impact of exercise intensity, exercise duration, body mass, and sweat loss on trapped sweat; and (3) trapped sweat during on‐court training to assess the ecological validity of the laboratory‐based results. Previous studies (Cheuvront et al. 2002; Noonan and Stachenfeld 2012; O'Neal et al. 2012) have shown that trapped sweat in clothing can lead to underestimations in sweat loss; however, this study is the first to measure trapped sweat in basketball uniforms and demonstrate that the magnitude of trapped sweat and the associated errors in sweat loss estimates are dependent upon common exercise/sport factors (exercise intensity, exercise duration, and sweat loss).

The main finding from this study was that trapped sweat in basketball uniforms varied considerably; ranging from 0 to 1.33 kg. Trapped sweat was associated with 0–32% underestimation in sweat loss during 150 min of laboratory‐based intermittent exercise and similar results were observed during the field‐based testing (0–1.27 kg trapped sweat; 0–36% underestimation in sweat loss). These results can help inform best practices in measuring sweat losses in the field. Clothed body mass measurements should be used with caution, as trapped sweat is highly variable. For instance TS can cause a significant underestimation in sweat loss in athletes with high sweating rates. In theory, a postexercise fluid replacement strategy based on a sweat loss that was underestimated by ~30% would translate to athletes starting their next training session with a ~0.8–1.5% fluid deficit (in players with a body mass of 84.1–118.8 kg; i.e., subjects in which a ~30% underestimation was observed in this study). In contrast when sweat loss is low, trapped sweat is also low (e.g., mean of 0.11 kg in the Low trial) and therefore has minimal effects on the accuracy of sweat loss estimates (8% underestimation) or fluid intake strategies (represents only 0.12% of body mass).

The trapped sweat literature in sport‐specific clothing/uniforms is relatively sparse. Two studies have measured trapped sweat in the clothing of distance runners (Cheuvront et al. 2002; O'Neal et al. 2012). Cheuvront et al. (2002) found that mean trapped sweat in female runners' clothing was 0.20 to 0.27 kg (across two environmental conditions) during a 30‐km treadmill run, which was similar to the mean trapped sweat during the Moderate intensity basketball‐simulation trial in this study (0.21 kg). The error introduced by trapped sweat was also comparable between studies; that is, 8–10% in running clothing (Cheuvront et al. 2002) versus 12% in basketball uniforms. O'Neal et al. (2012) reported that mean mass of sweat retained in shoes and clothing and toweled off skin after a ~1‐h outdoor run was 0.49 kg and 0.28 kg in men and women, respectively. In this study (O'Neal et al. 2012), the percentage of sweat loss trapped in the runner's clothing was higher (26% for men and 22% for women) than that of Cheuvront et al. (2002) or this study, perhaps due in part to the higher relative humidity (mean of 86 and 72% during morning and evening runs), which could have limited the rate of sweat evaporation. One study has measured sweat retained in ice hockey uniforms during a laboratory‐based hockey game simulation (Noonan and Stachenfeld 2012). Not surprisingly, mean trapped sweat in ice hockey uniforms (0.47–0.59 kg across fabric conditions) was higher than basketball clothing and also accounted for a greater mean percentage of total sweat loss (32–38%) (Noonan and Stachenfeld 2012).

A point of difference between studies was that trapped sweat was more variable in basketball uniforms compared with that of running clothing (Cheuvront et al. 2002; O'Neal et al. 2012) and the ice hockey uniforms (Noonan and Stachenfeld 2012). For example, the interindividual range in the treadmill runners' trapped sweat was 0.03–0.66 kg (Cheuvront et al. 2002), whereas basketball players' trapped sweat varied from 0.00 to 1.33 kg (across trials). This is likely due, at least in part, to the wider range in sweat loss experienced by the basketball players (0.39–4.18 kg) than the runners (1.31–3.88 kg) (Cheuvront et al. 2002). This in turn is likely due to the large range of body mass and the three different exercise intensities used herein. The results shown in both Table 2 (correlation matrix) and Figure 3 (linear regressions) buttress this interpretation. Also of note from Figure 3, is the observation of no trapped sweat in three subjects whom were on the lower ends of the ranges for body mass (57.79–63.11 kg) and sweat loss (0.39–1.01 kg) in the laboratory study. In contrast, the outlier who experienced the highest amount of trapped sweat in the laboratory (0.75–1.33 kg), was by far the largest subject (118.8 kg) and experienced the highest volume of sweat loss (3.50–4.18 kg across trials). Both observations are in agreement with the correlation and regression results. It is also important to note that, although the largest subject is considered an outlier in the laboratory dataset, his results fell within the range of the 11 elite (college and professional) players in the on‐court dataset with respect to body mass (83.00–126.55 kg), sweat loss (1.06–3.86 kg), and trapped sweat (0.00–1.27 kg).

As expected, sweat losses and trapped sweat increased with an elevation in exercise intensity (Table 1). The percent contribution of trapped sweat and nonsweat sources to the error in sweat loss calculations also varied with exercise intensity (Fig. 1). With an increase in intensity, the error due to nonsweat sources decreased and the error due to trapped sweat increased. Interestingly, during the High trial the underestimation in sweat loss due to trapped sweat essentially cancelled out the overestimation due to respiratory water loss and metabolic mass loss. This supports the notion that trapped sweat is offset by nonsweat losses under some circumstances, as suggested by Cheuvront and Haymes (2001), but is dependent on duration and intensity and is highly variable. Another factor that impacted trapped sweat was the duration of exercise. During exercise where sweating efficiency is less than 100%, clothing mass increases until the sweat content of clothing reaches equilibrium between absorption rate into clothing and evaporation rate from wetted clothing surface. In this study, this point of equilibrium was reached at ~60 min of the Low and Moderate trials and ~90 min of the High trials (Fig. 2). These results regarding trapped sweat in uniforms could be helpful in determining sweat efficiency to inform sweat prediction and heat strain models (Kubota et al. 2014) in basketball and comparable team sports.

Previous studies on trapped sweat have determined that clothing garments comprised of cotton retains significantly more sweat than synthetic fabric (Gavin et al. 2001; Brazaitis et al. 2010; Noonan and Stachenfeld 2012; de Sousa et al. 2014; Hooper et al. 2015). Accordingly, most sports uniforms are made of synthetic materials to promote evaporation of sweat (i.e., moisture‐wicking fabrics), and therefore the basketball uniform used in this study was comprised primarily of nylon and polyester. In addition, to be consistent with current trends in sports clothing, the uniform included compression clothing, which were also made of synthetic fabric.

In this study a laboratory‐based protocol was used so that independent variables, such as environment and clothing ensemble could be standardized, and exercise intensity could be systematically controlled to determine its impact on trapped sweat. The Low, Moderate, and High basketball‐simulation trials achieved three distinct progressive levels of intensity, based on HR, RPE, and VAS ratings of muscle fatigue, tiredness, effort, and physical demand. The mean HR and RPE observed during the High trial were similar to that reported during basketball scrimmages and games in previous investigations (McInnes et al. 1995; Narazaki et al. 2009). For instance, Narazaki et al. (2009) reported a mean HR of 169 bpm (88% of HR_max_) and mean RPE of 13.7 in male collegiate basketball players (20.8 ± 1.0 years, 91.9 ± 17.5 kg) during a scrimmage. To date there is limited information available regarding the physiological demands of and subjective responses to basketball practice sessions. However, one study has measured sweat loss in basketball players during collegiate practices. Thigpen et al. (2014) reported sweat losses (without correction for RWL and MML) of 2.47 L (0.87 L/h) during a 170‐min sport‐specific practice and 0.97 L (1.26 L/h) during a 45‐min conditioning practice in Division II college men. By comparison, partially corrected sweat losses during the 150‐min Moderate (1.93 L or 0.77 L/h) and High (2.51 L or 1.00 L/h) trials in this study were similar to the sweat losses reported in Thigpen et al. (2014), suggesting that our protocol was representative of actual practices. The ecological validity of the laboratory‐based protocol was also supported by the comparable trapped sweat observed in basketball clothing of players during on‐court practice sessions. However, future research is needed to determine trapped sweat in basketball uniforms during competition.

## Conclusions

Trapped sweat, and the impact it had on subjects' sweat loss estimates, during intermittent basketball‐simulated exercise varied considerably. Increased trapped sweat in basketball uniforms was associated with higher sweat loss, which was in turn directly related to exercise intensity. The results of this study can help inform best practices in field‐based measurements of sweat losses: athletes should be weighed while nude or wearing minimal clothing (compression shorts) to limit errors in sweat loss estimates associated with trapped sweat in uniforms. However, more work is needed to determine trapped sweat in uniforms during competition. Additional future avenues of research include measuring trapped sweat in team sports that (1) are played outdoors in variable environmental conditions (e.g., soccer, tennis, baseball) to determine the effects of air temperature, relative humidity, and wind; and (2) require more clothing or protective equipment (e.g., American football).

## Conflict of Interest

The authors are employed by PepsiCo, Inc. The views expressed in this article are those of the authors and do not necessarily reflect the position or policy of PepsiCo, Inc.
